# Antioxidant Capacity, Enzyme Activities Related to Energy Metabolism, and Transcriptome Analysis of *Crassostrea hongkongensis* Exposed to Hypoxia

**DOI:** 10.3390/antiox13091063

**Published:** 2024-08-30

**Authors:** Pingping He, Wei Li, Pinyuan Wei, Linyuan Jiang, Junliang Guan, Yuan Ma, Li Zhang, Yongxian Chen, Yusi Zheng, Xingzhi Zhang, Jinxia Peng

**Affiliations:** Guangxi Key Laboratory of Aquatic Genetic Breedingand Healthy Aquaculture, China (Guangxi)-ASEAN Key Laboratory of Comprehensive Exploitation and Utilization of Aquatic Germplasm Resources, Ministry of Agriculture and Rural Affairs, Guangxi Academy of Fishery Sciences, Nanning 530021, China; hpp140206@foxmail.com (P.H.); lw1397375050@foxmail.com (W.L.); weipinyuan@hotmail.com (P.W.); jly253346541@foxmail.com (L.J.); gjl524519263@foxmail.com (J.G.); ma1039135848@foxmail.com (Y.M.); arbily0403@foxmail.com (L.Z.); yohnsonchen@foxtail.com (Y.C.); yuaihuaji@foxmail.com (Y.Z.)

**Keywords:** transcriptome, *Crassostrea hongkongensis*, hypoxia stress, RT-qPCR, T-AOC, glycogen content, enzyme activities

## Abstract

*Crassostrea hongkongensis* (*C. hongkongensis*) is one of the three most commonly cultivated oyster species in China. Seasonal hypoxia is one of the most serious threats to its metabolism, reproductive behavior, and survival. To investigate the effects of hypoxia stress on the antioxidant capacity and energy metabolism of *C. hongkongensis*, the total antioxidant capacity (T-AOC), glycogen content, and enzyme activities (phosphofructokinase, PFK; pyruvate kinase, PK; phosphoenolpyruvate carboxykinase, PEPCK) of oysters were determined under normoxic (DO 6 ± 0.2 mg/L) and hypoxic (DO 1.5 mg/L) conditions at 0 h, 6 h, 48 h, and 72 h. We also determined the T-AOC, glycogen content, and enzyme activities of oysters under reoxygenation (recovered to normoxia for 24 h). To further examine the potential molecular regulatory mechanism of hypoxic adaptation, a transcriptome analysis was conducted on the gill of *C. hongkongensis* under normoxia (N, 72 h), hypoxia (H, 72 h), and reoxygenation (R). After being exposed to hypoxia for 6 h, the T-AOC, glycogen content, and enzyme activities of PK, PFK, and PEPCK in *C. hongkongensis* were significantly decreased. However, after prolonging the duration of hypoxia exposure for 72 h, the T-AOC, glycogen content, and enzyme activities increased compared to that of 48 h. After 24 h reoxygenation, the T-AOC, glycogen content, and enzyme activity of PK and PFK returned to close to initial levels. In addition, a transcriptome analysis discovered 6097 novel genes by mapping the *C. hongkongensis* genome with the clean reads. In total, 352 differentially expressed genes (DEGs) were identified in the H vs. N comparison group (235 upregulated and 117 downregulated genes). After recovery to normoxia, 292 DEGs (134 upregulated and 158 downregulated genes) were identified in the R vs. N comparison group, and 632 DEGs were identified (253 upregulated and 379 downregulated genes) in the R vs. H comparison group. The DEGs included some hypoxia-tolerant genes, such as phosphoenolpyruvate carboxykinase (*PEPCK*), mitochondrial (*AOX*), tyramine beta-hydroxylase (*TBH*), superoxide dismutase (*SOD*), glutathione S-transferase (*GST*), and egl nine homolog 1 isoform X2 (*EGLN1*). Additionally, DEGs were significantly enriched in the KEGG pathways that are involved in hypoxia tolerance, including the metabolism of xenobiotics by cytochrome P450 pathways and the HIF-1 signaling pathway. Then, we selected the five hypoxic-tolerant candidate DEGs for real-time quantitative polymerase chain reaction (RT-qPCR) validation, and the results were consistent with the transcriptome sequencing data. These discoveries have increased our understanding of hypoxia tolerance, recovery ability after reoxygenation, and molecular mechanisms governing the responses to hypoxia in *C. hongkongensis*.

## 1. Introduction

The amount of inorganic salts and organic matter input into an estuary increases sharply as a result of human activity, and this induces seasonal hypoxia in the estuary area and adjacent sea areas [1,2]. Hypoxia refers to a dissolved oxygen (DO) level of less than 2.0 mg/L in a marine environment [3], which affects organisms’ movement, metabolism, and reproductive behavior, and can even lead to death [4]. Therefore, mollusks inhabiting estuarine areas often face the threat of a hypoxic environment because of their poor mobility [5].

*Crassostrea hongkongensis* (*C. hongkongensis*) is one of the three most commonly cultivated oyster species with economic importance, distributed throughout the South China Sea [6]. *C. hongkongensis* is a sessile bivalve inhabiting the intertidal zone. The oyster is distributed in coastal estuaries in the south of China, where human activities are intense, and industry and agriculture are concentrated [7]. Thus, *C. hongkongensis*, an intertidal sessile model species, may suffer from hypoxia during low tides. One study showed that during hot and foggy weather in July and August, the DO in seawater decreased by 45.5% (DO 1.94 mg/L) in the Maowei Sea, Qinzhou, China, which led to the mortality rate of *C. hongkongensis* reaching 60%; while utilizing the oxygenation function of an aerator, the mortality of *C. hongkongensis* significantly reduced. The results indicated that low oxygen has become an important environmental factor restricting marine aquaculture [2].

Shellfish generally have well-developed hypoxia tolerance, through responses such as actively reducing their own metabolic rate when facing hypoxia stress [8]. In a hypoxic environment, the feeding rate and excretion rate of *Ruditapes decussatus* decreased, along with its energy metabolism rate [9]; *Crassostrea virginica* limited the activity of metabolic enzymes through protein phosphorylation modifications, thereby reducing its basal metabolic rate [10]. The exposure to hypoxia is always relevant to the change in the antioxidant and enzyme activities related to energy metabolism in bivalves. Wei et al. examined the hypoxia stress enzyme activities of *C. hongkongensis*, such as superoxide dismutase (SOD) and catalase (CAT) [11]. Exposed to hypoxia, the kinetic properties of both phosphofructokinase (PFK) and pyruvate kinase (PK) changed in *Littorina littorea* [12], and the level of phosphoenolpyruvate carboxykinase (PEPCK) increased in *Crassostrea gigas* [13]. Transcriptome sequencing has been widely used for understanding the molecular mechanisms of shellfish against environmental stress, including metal stress, temperature stress, and salinity stress [14,15,16]. Some reports have noted the effects of hypoxia stress on the metabolism and transcriptional regulation in bivalves. A transcriptomic analysis related to the regulation of *Mytilus edulis* exposed to acute hypoxia has been conducted [17]. Yang et al. performed an integrated analysis of transcriptomics and metabolomics to reveal the response of *Pinctada fucata martensii* to long-term hypoxia [18]. To date, data on hypoxia-responsive transcriptomics in *C. hongkongensis* remain limited.

Little is known about the tolerance of *C. hongkongensis* to hypoxia. Therefore, in this study, the goal was to evaluate the antioxidant response, energy metabolism, and transcriptomic response of *C. hongkongensis* under hypoxic stress, as well as to gain knowledge concerning how *C. hongkongensis* initiates a response to low oxygen levels. We integrated analyses of antioxidant capacity and enzyme activities related to energy metabolism in normoxic, hypoxic, and reoxygenated conditions with a transcriptomic analysis to identify the differentially expressed genes and the KEGG pathways related to the hypoxic response. These data enhance our understanding of the mechanisms governing the responses to hypoxia in *C. hongkongensis*.

## 2. Materials and Methods

### 2.1. Hypoxia Experiment

Two-year-old *C. hongkongensis* specimens were obtained from an oyster-breeding area in Lianzhou Bay (Beihai, China), where the conditions were as follows: temperature at 30 ± 2 °C, salinity 20 ± 1‰, dissolved oxygen (DO) 5.6 ± 0.2 mg/L, and pH 7.8 ± 0.2. These oysters had a mean body weight of 89.07 ± 10.47 g, mean shell height of 87.62 ± 7.66 mm, and mean shell length of 50.30 ± 4.40 mm. The experiment was performed in the laboratory of the Beihai aquaculture base of the Guangxi Academy of Fishery Sciences (E109°7′35″, N21°29′13″). Before the hypoxia experiment, oysters were reared at an average temperature of 30 °C and a salinity of 22‰ for one week. Oysters were fed with algal liquid (*Dicrateria* sp, *Chaetoceros*, and *Platymonas* mix) in the morning and evening.

**Figure 1:**
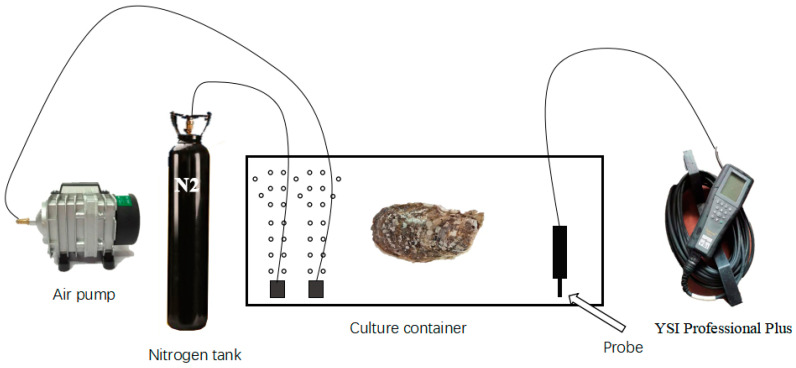
The experimental hypoxia measurement device.

Based on our team’s previous research data regarding hypoxia, we selected 120 experimental oysters and placed oysters into normoxia (DO 6.0 ± 0.2 mg/L) and hypoxia (DO 1.5 ± 0.2 mg/L) conditions. The 120 oysters were evenly distributed among six 500 L tanks of filtered seawater and were evenly divided into control and experimental groups. After 72 h of hypoxic treatment, the conditions were recovered to normoxia for 24 h (reoxygenation DO 6.0 ± 0.2 mg/L). Normoxic conditions were maintained by continuously supplying oxygen to the seawater, and hypoxic conditions were maintained by supplying nitrogen to the seawater [10]. The air and nitrogen aeration rates of containers were individually controlled through a gas flow meter to maintain the concentration of dissolved oxygen. A YSI Professional Plus (YSI, Yellow Springs, OH, USA) was used as the dissolved oxygen detection instrument (Figure 1). To avoid the exchange of oxygen between the seawater and air, we sealed the buckets containing seawater with plastic film and rubber bands after the DO was reduced to the target concentration. The DO of the aquaculture seawater was detected using YSI Professional Plus.

### 2.2. Antioxidant Capacity and Enzyme Activity Determination

The gill and adductor muscle tissue of two alive oysters from each tank (six tanks) were collected at time points (0 h, 6 h, 48 h, and 72 h hypoxia, as well as reoxygenation). A total of 60 oysters were sampled, and all samples were stored at −80 °C. The total antioxidant capacity (T-AOC), glycogen content, and enzyme activities of PFK, PK, and PEPCK were determined. T-AOC was determined using oyster gill tissue, following the method of Zhang et al. [19], with three replicates per time point (0 h, 6 h, 48 h, 72 h, and 96 h), while glycogen content, PFK enzyme activity, PK enzyme activity, and PEPCK enzyme activity were determined using adductor muscle, with three replicates per time point. T-AOC, glycogen content, PFK enzyme activity, PK enzyme activity, and PEPCK enzyme activity were determined according to the instructions of the reagent kit (T-AOC: D799276-0100, glycogen content: D799398-0100, PFK: D799442-0100, PK: D799444-0100, Sangon Biotechnology Co., Ltd., Shanghai, China; PEPCK: G0830W, Grace Biotechnology Co., Ltd., Suzhou, China). A total of 30 oysters (60 tissue samples) in the control and experimental groups were used for antioxidant capacity and enzyme activity determination.

The experimental data were subjected to a one-way ANOVA and Duncan’s test using SPSS 23.0 software, with a significance level of 0.05 (*p* < 0.05) for data statistics. The software Excel 2013 was used for plotting.

### 2.3. RNA Extraction and Sequencing

We selected three oysters at the 72 h normoxic condition, 72 h hypoxic condition, and 24 h after recovery to the normoxic condition, respectively. The gill tissue of nine oysters was sampled. For each sampled individual, a portion of the gill tissue was immediately frozen in liquid nitrogen for RNA sequencing and a portion immersed in RNAlater (QianGen, Hilden, Germany) for real-time quantitative polymerase chain reaction (RT-qPCR) analysis. Then, all samples were stored at −80 °C. Total RNA was extracted from the nine gill samples using TRI Reagent Solution TR118 (Ambion, Austin, TX, USA). The separated RNA concentrations and purity were measured with NanoDrop 2000 (Thermo Fisher Scientific, Wilmington, DE, USA). We determined RNA integrity with the Agilent Bioanalyzer 2100 system (Agilent Technologies, Santa Clara, CA, USA). The cDNA libraries were sequenced on an Illumina Novaseq 6000 platform (NEB, Ipswich, MA, USA).

### 2.4. Sequence Processing and Functional Annotation

Raw data were first processed by removing adapters and sequences containing poly-N, and low-quality reads were excluded from the raw reads to obtain clean data. We subsequently calculated the Q30 values and guanine-cytosine (GC)-content levels of the clean data. The clean data were then compared with the reference genome (CNGB BioProject number PRJCA013250) with Hisat2 (2.0.4). To optimize the annotation information of a genome, we achieved the discovery of novel transcripts and genes by StringTie on the basis of the reference genome [20]. The mapped reads were compared with the annotations of the genome. The transcript regions without annotation were defined as novel transcripts. Novel genes were annotated by DIAMOND against several databases, including NR, Swiss-Prot, KOG, COG, GO, and KEGG databases [21,22,23,24,25,26].

### 2.5. Analysis of DEGs

To identify DEGs under normoxic, hypoxic, and reoxygenation conditions, we estimated the quantification of gene expression level by fragments per kilobase of transcript per million fragments mapped (FPKM). The DEGs among the three libraries were distinguished with DEseq2 software. We determined the threshold of the *p*-value with the false-discovery rate (FDR). In this study, genes with an FDR ≤ 0.05 and fold change ≥ 2 were identified as DEGs.

In addition, we conducted GO and KEGG enrichment analyses for DEGs. GO enrichment analysis of the DEGs was carried out with the GOseq R packages [27]. The KOBAS program [28] was used to detect the enrichment of DEGs in KEGG pathways.

### 2.6. Real-Time Quantitative Polymerase Chain Reaction (RT-qPCR)

To validate the expression trends of DEGs under the hypoxic and reoxygenation conditions of *C. hongkongensis*, we selected five significant DEGs for qPCR. The primer sequences of two hypoxic downregulated and three hypoxic upregulated genes are listed in Table 1. We used *GAPDH* as the internal reference gene. We conducted qPCR on an Applied Biosystems StepOnePlusTM Real-Time System with SYBR Select Master Mix (ABI, Los Angeles, CA, USA). The RT-qPCR program was as follows: 30 s at 95 °C; then, proceed with 40 cycles for 10 s at 95 °C; and 30 s at 60 °C. Each sample was tested three times. The expression levels of genes were calculated by the 2^−ΔΔCT^ method. StepOne Software v2.3 and BioRadCFXManager (3.1) were used for data analysis.

## 3. Results

### 3.1. Antioxidant Capacity, Glycogen Content, and Enzyme Activities

**Figure 2:**
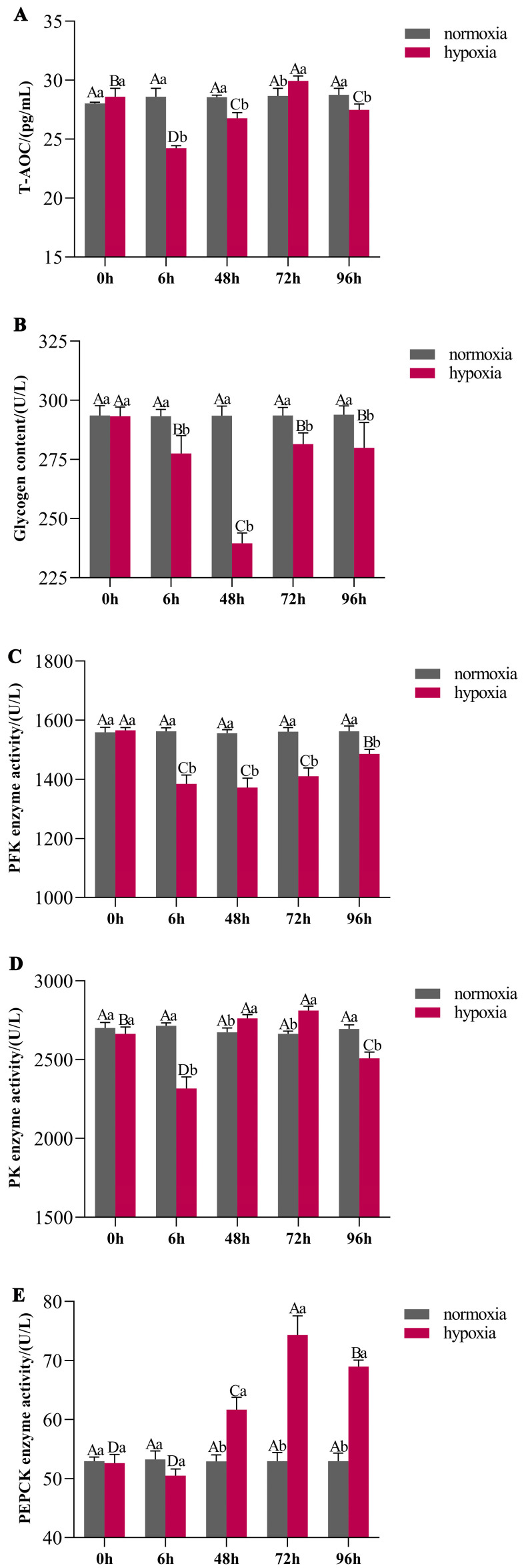
The total antioxidant capacity (T-AOC) (**A**), glycogen content (**B**), and enzyme activities of PFK (**C**), PK (**D**), and PEPCK (**E**) of *C. hongkongensis* exposed to 0 h, 6 h, 48 h, and 72 h hypoxic treatment, as well as 96 h treatment (72 h hypoxia + 24 h reoxygenation). The control group (normoxia) is represented by gray bars and the experimental groups (hypoxia) by purple. Means with the same letter have no significant differences (*p* > 0.05). The significance of the difference between treatments at the same time point is indicated by the lowercase letters a and b, and differences between different time points in the same treatment group are indicated by the capital letters A, B, C, and D (*p* < 0.05).

In the hypoxic treatment, the T-AOC was depressed at 6 h; at 72 h, the T-AOC increased, reaching the maximum value with a significant difference compared to the normoxic group (*p* < 0.05) (Figure 2A). After 24 h of reoxygenation, the T-AOC decreased. In the hypoxic treatment, the glycogen content showed a trend of first decreasing and then increasing (Figure 2B). After 6 h and 48 h of hypoxic treatment, the glycogen content decreased, and that of the hypoxia group was significantly less than that of the normoxic group (*p* < 0.05), while the glycogen content during 48 h to 72 h of hypoxia and 24 h of reoxygenation significantly increased compared to that at 48 h hypoxia (*p* < 0.05) (Figure 2B).

Exposed to hypoxic stress, the PFK (Figure 2C), PK (Figure 2D), and PEPCK (Figure 2E) enzyme activity of *C. hongkongensis* decreased at 6 h; the PFK enzyme activity of oysters exposed to 48 h hypoxia decreased compared to that of oysters exposed to 6 h, but the PK and PEPCK enzyme activity significantly increased. The PK and PEPCK enzyme activity of *C. hongkongensis* exposed to 72 h hypoxia was significantly higher than normoxia (*p* < 0.05), while PFK was lower. After 24 h reoxygenation, the PEPCK enzyme activity increased significantly compared to normoxia, while the PFK and PK enzyme activity was significantly depressed (*p* < 0.05).

### 3.2. Sequencing, Sequence Assembly, and Annotation

In total, we constructed nine gill libraries, including normoxic (N), hypoxic (H), and reoxygenation (R) conditions of *C. hongkongensis* (each condition consisting of three libraries). After quality control, 197,812,420 clean reads remained from nine libraries. The GC content of the clean data exceeded 40%, and the Q30 percentage ranged from 95.26% to 95.75% (Table 2). The clean reads can be obtained from the National Center for Biotechnology Information (NCBI) Sequence Read Archive (SRA; submission number SUB12304423). The clean reads were mapped to the *C. hongkongensis* genome with Hisat2, and the mapped reads exceeded 80.29%. We assembled the mapped reads using StringTie software (1.3.4d) [20] and compared the data with the original annotations of the genome. Eliminating short transcripts or those containing only one exon, we discovered 6097 novel genes, including 2079 genes annotated: NR (1864), Swiss-Prot (83), KOG (126), COG (39), GO (852), and KEGG (640).

### 3.3. Candidate Genes under Hypoxic Conditions

**Figure 3:**
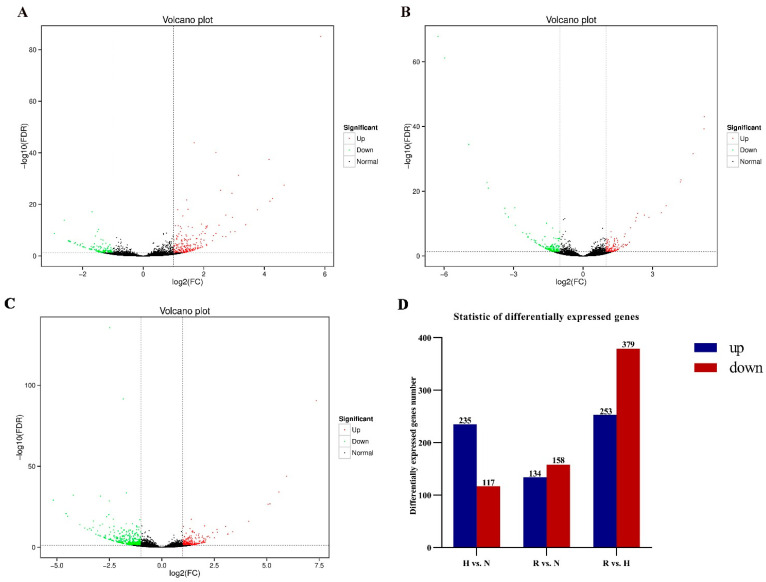
Indentification of DEG: (**A**) Volcano plot of DEGs in hypoxia (H) vs. normoxia (N); (**B**) Volcano plot of DEGs in reoxygenation (R) vs. normoxia (N); (**C**) Volcano plot of DEGs in reoxygenation (R) vs. hypoxia (H). Red: upregulated genes; Green: downregulated genes; (**D**) Statistic of DEGs of *C. hongkongensis*, the blue bar represents the number of up-regulatedDEGs, and the red bar represents the number of down-regulated DEGs.

We identified a total of 352 DEGs between the N and H libraries, including 235 hypoxia-upregulated and 117 hypoxia-downregulated genes (Figure 3A,D; Table S1). In total, 292 DEGs were obtained in the R vs. N library comparison, including 134 reoxygenation-upregulated genes and 158 reoxygenation-downregulated genes (Figure 3B,D; Table S2), and 632 DEGs were identified (253 upregulated and 379 downregulated genes) in the R vs. H comparison group (Figure 3C,D; Table S3).

In this study, we identified some known hypoxic-tolerant genes in the DEGs of *C. hongkongensis* (Table 3), including the transient receptor potential cation channel subfamily M member 2 (*TRPM2*, CH1000250), tyramine beta-hydroxylase (*TBH*, CH1001282), superoxide dismutase (*SOD*, CH0900166), alternative oxidase (*AOX*, CH0502591), and globin isoform X3 (CH0200562). These genes including *TRPM2*, *TBH*, and *SOD* were significant DEGs both in the H vs. N and R vs. N comparison groups, whereas *AOX* was differentially expressed in the H vs. N comparison group, and globin isoform X3 was differentially expressed in the H vs. N and R vs. H comparison groups. We speculated that they might be hypoxic-tolerant candidate genes in *C. hongkongensis*.

### 3.4. Functional Annotation of DEGs under Hypoxic Conditions in C. hongkongensis

**Figure 4:**
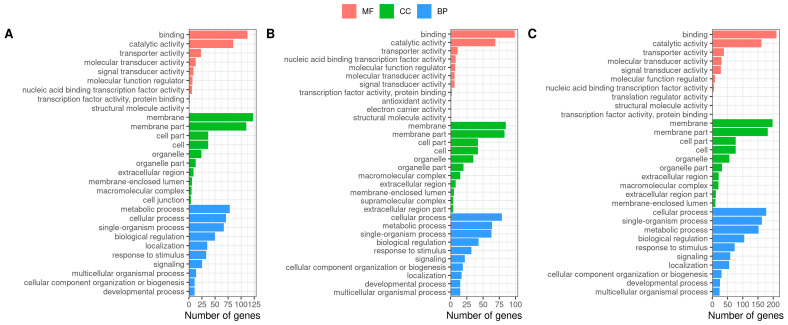
GO terms for DEGs: (**A**) hypoxia (H) vs. normoxia (N); (**B**) reoxygenation (R) vs. normoxia (N); (**C**) reoxygenation (R) vs. hypoxia (H).

We conducted a GO functional analysis of DEGs. The results of the three comparison groups were consistent. In the biological process term, the DEGs were associated with the cellular and metabolic process. In the cellular component term, the DEGs were involved in the membrane and membrane parts. In the molecular function term, the DEGs were related to the functional categories of binding and molecular transducer activity (Figure 4).

**Figure 5:**
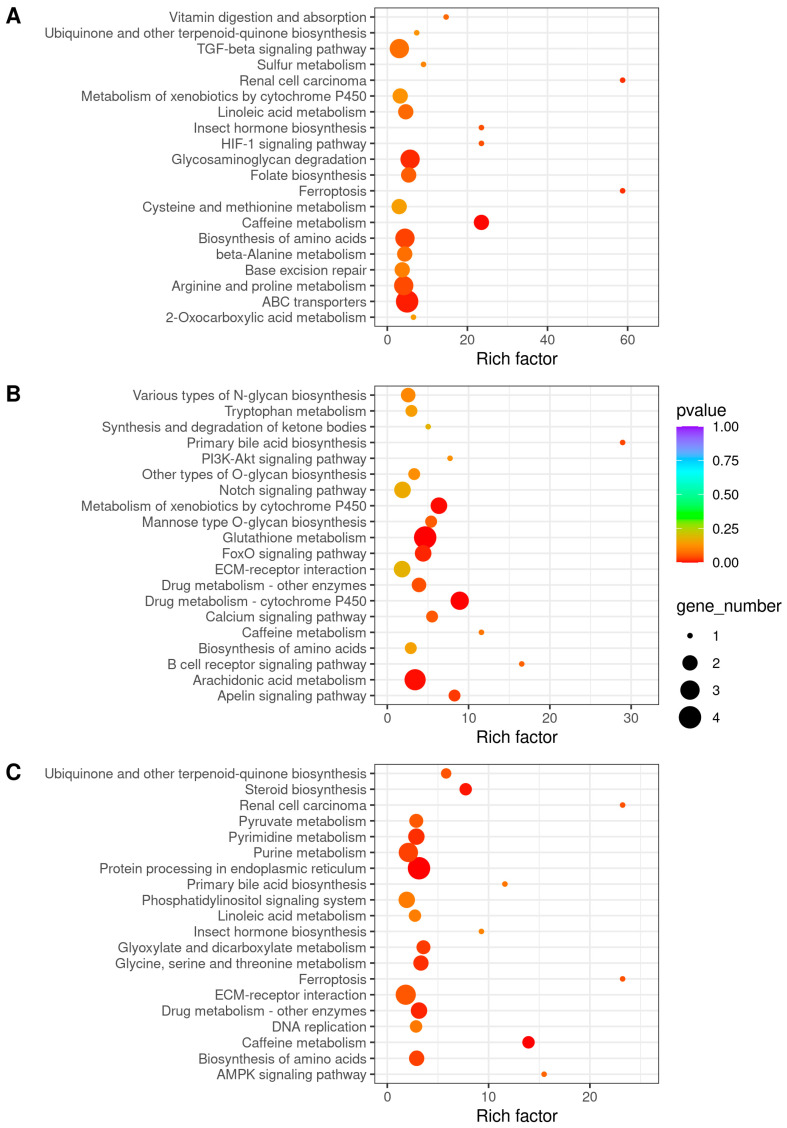
Top-20 significantly enriched KEGG pathways: (**A**) hypoxia (H) vs. normoxia (N); (**B**) reoxygenation (R) vs. normoxia (N); (**C**) reoxygenation (R) vs. hypoxia (H).

In the top-20 enriched KEGG pathways, the biosynthesis of amino acids pathway, the ubiquinone and other terpenoid-quinone biosynthesis pathway, and the caffeine metabolism pathway were significantly enriched in all three comparison groups. The metabolism of xenobiotics by cytochrome P450 pathway was significantly enriched in both the H vs. N and R vs. N comparison groups (Figure 5). And several pathways were enriched in only one comparison group, such as the HIF-1 signaling pathway, which was only enriched in the H vs. N comparison group.

### 3.5. KEGG Pathway Network Enriched by DEGs

**Figure 6:**
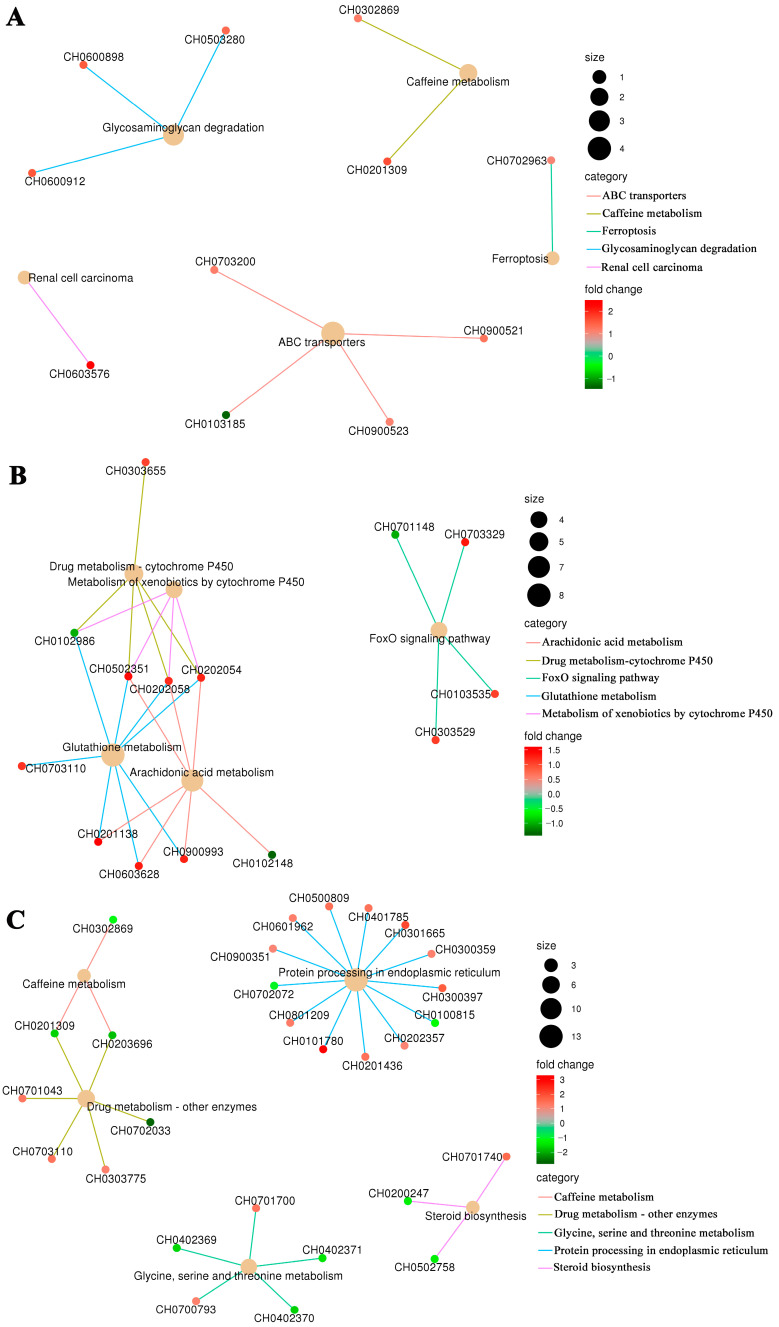
KEGG pathway enrichment network of DEGs in the H vs. N (**A**), R vs. N (**B**), and R vs. H (**C**) comparison groups.

To study the correlations between DEGs and KEGG pathways, five pathways with the lowest Q-values in the H vs. N, R vs. N, and R vs. H comparison groups were identified, respectively. Of these, several pathways were enriched in the same DEGs. In the H vs. N comparison, the five pathways were not enriched with common DEGs (Figure 6A). In the R vs. N comparison, the upregulated DEGs including glutathione S-transferase (CH0502351), S-crystallin SL11 (CH0202054), and S-crystallin SL11-like (CH0202058), working synergistically to perform biological functions in the four pathways of drug metabolism-cytochrome P450, metabolism of xenobiotics by cytochrome P450, glutathione metabolism, and arachidonic acid metabolism (Figure 6B). In the R vs. H comparison, the downregulated DEGs of xanthine dehydrogenase/oxidase-like (CH0201309, CH0203696) played a role in regulating the caffeine metabolism pathway and drug metabolism-other enzymes pathway (Figure 6C).

### 3.6. RT-qPCR Confirmation of DEGs

**Figure 7:**
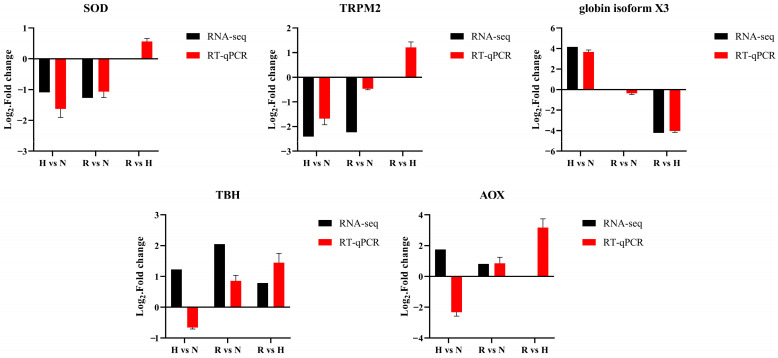
Comparison of DEG expression data between RNA-seq (black bars) and RT-qPCR (red bars). The *x*-axis presents the comparison groups, and the *y*-axis presents the fold change in DEG expression.

We selected five hypoxic-tolerant candidate genes from transcriptome sequencing data for RT-qPCR validation. The RT-qPCR results of most DEGs were consistent with the RNA sequencing (Figure 7). The results confirmed the credibility of the transcriptome sequencing data.

## 4. Discussion

Hypoxia is a common phenomenon for aquatic organisms [29,30,31]. To adapt to low-oxygen environments, shellfish spontaneously regulate their physiological response processes, including their respiration, protein regulation, oxidative metabolism, and immune system [32,33]. Thus, for this study, T-AOC, glycogen content, and the enzyme activities of *C. hongkongensis* exposed to hypoxia were determined, and the gill of *C. hongkongensis* was selected as the source of tissue for transcriptome analysis of the response to hypoxia.

T-AOC is a comprehensive indicator used to determine the antioxidant level of organisms [34]. Glycogen is one of the main energy sources of organisms and is closely related to energy metabolism. In the hypoxia group, the T-AOC and glycogen content of *C. hongkongensis* decreased at 6 h. As the duration of hypoxia prolonged, the T-AOC and glycogen content gradually increased. At 72 h, the T-AOC and glycogen content were significantly higher than at 48 h hypoxia (*p* < 0.05). After recovering to normoxia for 24 h, the T-AOC and glycogen content of C. *hongkongensis* exposed to hypoxia returned to normal levels. Under hypoxic conditions, T-AOC may display compensatory increases to regulate the antioxidant levels of *C. hongkongensis*. Glycogen, as a carbohydrate, is involved in catabolism via glycolysis to produce ATP [35]. The compensatory increase in ATP maintained the normal turnover needs in the organism.

Most marine invertebrates exposed to environmental stress will autonomously transfer energy metabolism toward glycolysis [36,37,38]. PFK, PK, and PEPCK, as important enzymes, play important roles in glycolysis. PFK is a metabolic enzyme that determines carbohydrate utilization, and the enzymes PK and PEPCK catalyze phosphoenolpyruvate (PEP) to produce ATP [35]. Under 6 h of hypoxia stimulation, the enzyme activities of PK, PFK, and PEPCK in *C. hongkongensis* rapidly decreased, indicating that the oysters immediately responded to hypoxia and altered their mode of energy metabolism. As the hypoxia duration prolonged, the enzyme activities of PK, PFK, and PEPCK gradually increased, which is generally consistent with the trend of glycogen content changing. These results indicated that *C. hongkongensis* had altered its metabolic process to anaerobic glycolysis, consistent with previous research. The PEP catabolism is accomplished via the catalysis of PK and PEPCK, which then further generates ATP for energy supply.

Under hypoxia, the expression levels of antioxidant genes and glycolysis-related genes may change, such as the *SOD* gene of *C. gigas* being significantly upregulated [39], and the expression levels of genes *HK*, *PEPCK*, and *UCP2* increasing [40]. In this study, we identified several known hypoxia-tolerance genes in the DEGs of *C. hongkongensis* by transcriptome sequencing, including *PEPCK*, *AOX*, *TBH*, and *SOD*. In the R vs. H comparison groups, the *PEPCK* gene was upregulated under hypoxia, while the expression level decreased during reoxygenation. The mRNA levels and enzyme activity of PEPCK increased in *C. hongkongensis* exposed to hypoxia, indicating that PEPCK regulated the mechanism of hypoxia tolerance.

Under hypoxia, the enzyme activity and mRNA levels of AOX in juvenile ark shells (*Anadara broughtonii*) increased [41]. In freshwater bivalves, *AOX* was strongly upregulated during anoxia in the gills [42]. A previous study showed that *AOX* modulated during hypoxic stress in the brains of Atlantic croaker [43]. *AOX* expression played an essential role in avoiding hypoxia-induced superoxide and H_2_O_2_ levels [44]. In the RT-qPCR results of our study, the expression of the *AOX* gene in *C. hongkongensis* gill was downregulated under hypoxia, opposite to previous research. This finding maybe indicated the negative regulation of the *AOX* gene in *C. hongkongensis* against hypoxia.

Octopamine (*OA*) is an important neuroactive substance that regulates several physiological functions and behaviors of many invertebrate species. *TBH* promotes the last step in OA biosynthesis. *TBH* is considered as a biomarker of stress in insects [45]. Therefore, *TBH* catalyzes the conversion of tyramine to OA [46], a process critical for stress responses. In *L. vanname*, expression of the gene *TBH* was upregulated after a low-temperature and Vibrio alginolyticus challenge [47]. In the RT-qPCR results of our study, the gene *TBH* in *C. hongkongensis* gill was downregulated under hypoxia; after recovering to normoxia for 24 h, the expression level increased, indicating the crucial role of *TBH* in orchestrating hypoxic responses.

Hypoxia and reoxygenation are physiological stressors that result in an overproduction of reactive oxygen species (ROS) and oxidative stress [48]. The most common ROS are superoxide radicals that are converted to hydrogen peroxide and oxygen by the enzyme SOD; catalase and glutathione peroxidase then degrade hydrogen peroxide to oxygen and water [49]. It has been reported that the injury by hypoxia or hypoxia/reoxygenation could be prevented by *SOD* [50]. In *Mytilus galloprovincialis*, expression of *SOD* was upregulated after 72 h of hypoxia and returned to the normoxic level after 24 h of reoxygenation [51]. Under hypoxic conditions, the expressions levels of the *SOD* gene and the activity of the antioxidant enzyme (SOD) were upregulated compared to the control group in hybrid yellow catfish [52]. The RT-qPCR results of our study demonstrated that the *SOD* gene in the gill of *C. hongkongensis* was downregulated under hypoxic conditions, a result that was the opposite of previous research results. This finding may suggest the presence of a negative regulating hypoxic adaptation.

DEGs were enriched in the KEGG pathways; both coregulated the metabolic network and the molecular mechanisms of the organisms. In this study, the transcriptome analysis of *C. hongkongensis* gill responding to hypoxia indicated that the metabolism of xenobiotics by cytochrome P450 pathway and HIF-1 signaling pathway may be associated with hypoxia-related responses.

The DEGs of the S-crystallin family (S-crystallin SL11, S-crystallin SL11-like) and the glutathione S-transferase family (glutathione S-transferase, glutathione S-transferase P 1 isoform X1, *GSTs*) were related with the enzyme glutathione S-transferase of the metabolism of xenobiotics by cytochrome P450 pathway (Table 4). S-crystallin is involved in the evolution of glutathione S-transferase [53]. *GST*, as a cell housekeeping gene engaged in the detoxification of xenobiotics, is related to the stress response [54]. It has been observed that the *GST* genes of silver sillago (*Sillago sihama*) were differentially expressed under hypoxia, and the metabolism of xenobiotics by cytochrome P450 pathway was concerning to the hypoxic response [55]. In this study, the expression levels of the *GST* family were upregulated or downregulated according to the changes in oxygen concentration, thereby regulating the metabolism of xenobiotics by cytochrome P450 pathway involved in the response to hypoxia or reoxygenation.

The HIF-1 signaling pathway, enriched in the H vs. N comparison group (Figure 5A), is crucial for the maintenance of oxygen homeostasis in aquatic animals, such as R. *philippinarum*, *Siniperca chuatsi*, and *Paralichthys olivaceus* [56,57,58]. Under hypoxic stress, the HIF-1 signaling pathway in organisms is activated [59]. Hypoxia-inducible factors (HIFs) are the master regulator of the cellular response to hypoxic stress in the HIF-1 signaling pathway [60]. Under normoxia, hypoxia-inducible factor prolyl hydroxylase (PHD) recognizes hydroxyl and uses molecular oxygen as a substrate to degrade HIF-α by ubiquitin-mediated proteolysis (Table 4). Egl nine homolog 1 isoform X2 (*EGLN1*), also known as *PHD2*, was an oxygen sensor in the HIF-1 signaling pathway, targeting two HIF-1α proteins for degradation under normoxic conditions [61]. In this study, under hypoxia, the mRNA expression level of *EGLN1* was upregulated in *C. hongkongensis* and negatively correlated with oxygen concentration. In HIF-α degradation, nitric oxide (NO) as an inhibitor of PHD competes with O_2_ for combining to iron, resulting in a redistribution of intracellular oxygen that activates *EGLN1* and causes HIF degradation [62]. Therefore, the HIF-1 signaling pathway in *C. hongkongensis* under hypoxia was activated to promote an adaptive response.

## 5. Conclusions

In this study, hypoxia tolerance, recovery ability after reoxygenation, and molecular mechanisms governing the responses to hypoxia were investigated in *C. hongkongensis*. Hypoxia significantly affected the antioxidant capacity and enzyme activities related to the energy metabolism of *C. hongkongensis*. However, at 24 h after recovery to the normoxic condition, the T-AOC, glycogen content, and enzyme activities of PK and PFK returned to close to the initial level. The results indicate that 72 h hypoxia (DO 1.5 ± 0.2 mg/L) did not cause serious damage to *C. hongkongensis*, and its organism function can be restored after 24 h reoxygenation. Then, DEGs such as *PEPCK*, *AOX*, *TBH*, *SOD*, *GST*, and *EGLN1*, as well as two KEGG pathways involved in hypoxic stress including the metabolism of xenobiotics by cytochrome P450 pathway and HIF-1 signaling pathway, were identified as being involved in the response. The results revealed the molecular mechanisms involved in the responses of *C. hongkongensis* exposed to normoxia, hypoxia, and reoxygenation.

## Figures and Tables

**Table 1:** Primer sequences used in RT-qPCR.

mRNA		Sequence (5′–3′)	Length (bp)
CH1000250	Fwd	AAGGTGACTTCAACACATCCG	220
	Rev	CCTACTTGGTTTTGCTCGTTAT	
CH1001282	Fwd	AGAAATGGGGTCAAGGTCG	119
	Rev	TAGCAGGTTCAGGTAACATACAA	
CH0900166	Fwd	TCCACCATAGGGTCCACG	242
	Rev	CCATTTCCAAAGGTCCGTC	
CH0502591	Fwd	GGTTATTTAGAAGTGGCGTCA	102
	Rev	GTGTGTACCAGTCCTACTGTCCT	
CH0200562	Fwd	CAACAATAGAGAGATTCCCGAT	210
	Rev	CTACCTTAAAGTCCTCCGAGC	
GAPDH	Fwd	GGATTGGCGTGGTGGTAGAG	184
	Rev	GTATGATGCCCCTTTGTTGAGTC	

**Table 2:** Sequencing data statistics.

Sample	Number of Clean Reads	Number of Clean Bases	GC (%)	% ≥ Q30
N1	25,206,800	7,511,666,510	40.8	95.56
N2	19,802,452	5,893,526,304	41.43	95.32
N3	20,465,606	6,087,523,020	41.01	95.31
H1	20,647,843	6,150,256,584	41.19	95.32
H2	24,095,305	7,170,772,744	41.7	95.47
H3	24,502,902	7,288,089,524	41.72	95.65
R1	20,244,717	6,033,282,358	41.11	95.45
R2	21,361,892	6,362,961,796	40.9	95.26
R3	21,484,903	6,389,008,412	41.5	95.75

**Table 3:** Differentially expressed genes involved in hypoxia tolerance (“-” means Log_2_.Fold change <1 or DEGs not expressed).

ID	Gene Description	Log_2_.Fold Change (H/N)	Log_2_.Fold Change (R/N)	Log_2_.Fold Change (R/H)
CH0900239	cytochrome P450 3A11 isoform X2	2.07	-	−1.08
CH0603576	egl nine homolog 1 isoform X2 (*EGLN1*)	2.4	-	−2.50
CH1000250	transient receptor potential cation channel subfamily M member 2 (*TRPM2*)	−2.41	−2.23	-
CH1001282	tyramine beta-hydroxylase (*TBH*)	1.23	2.05	-
CH0900166	superoxide dismutase (*SOD*)	−1.09	−1.27	-
CH0502591	alternative oxidase, mitochondrial (*AOX*)	1.75	-	-
CH0200562	globin isoform X3	4.16	-	−4.22
CH0502351	glutathione S-transferase (*GST*)	-	1.39	1.95
CH0202054	S-crystallin SL11	1.21	1.24	-
CH0202058	S-crystallin SL11-like	-	1.19	-
CH0102986	glutathione S-transferase P 1 isoform X1 (*GST*)	-	−1.01	-
CH0403890	Phosphoenolpyruvate carboxykinase (*PEPCK*)	-	-	−1.05
CH0301448	methionine adenosyltransferase 2 subunit beta (*MAT2A*)	−1.38	-	-
CH0900455	asparagine synthetase (*ASNS*)	1.55	-	
CH1000346	cytoplasmic aconitate hydratase isoform X2	1.04	-	−1.52
CH0403169	carbonyl reductase (*CR*)	1.34	-	
CH0201309	xanthine dehydrogenase/oxidase-like	1.641	-	−1.65
CH0302869	uricase-like	1.18	-	−1.11
CH0303655	senecionine N-oxygenase (*SNO*)	-	1.03	-
CH0601326	xanthine dehydrogenase/oxidase	-	−1.33	-
CH0203696	xanthine dehydrogenase	-	-	−1.83

**Table 4:** Differentially expressed genes (DEGs) enriched in KEGG pathways in hypoxia (H) vs. normoxia (N), reoxygenation (R) vs. normoxia (N), reoxygenation (R) vs. hypoxia (H).

Pathway ID	Pathway Term	DEGs Name
	Hypoxia (H) vs. Normoxia (N)	
ko01230	biosynthesis of amino acids	*MAT2A*; *ASNS*; cytoplasmic aconitate hydratase isoform X2
ko00232	caffeine metabolism	xanthine dehydrogenase/oxidase-like; uricase-like
ko00980	metabolism of xenobiotics by cytochrome P450	S-crystallin SL11; *CR*
ko04066	HIF-1 signaling pathway	*EGLN1*
	Reoxygenation (R) vs. Normoxia (N)	
ko00982	drug metabolism-cytochrome P450	glutathione S-transferase P 1 isoform X1; S-crystallin SL11; S-crystallin SL11-like; *SNO*; glutathione S-transferase
ko00980	metabolism of xenobiotics by cytochrome P450	glutathione S-transferase P 1 isoform X1; S-crystallin SL11; S-crystallin SL11-like; glutathione S-transferase
ko00232	caffeine metabolism	xanthine dehydrogenase/oxidase
	Reoxygenation (R) vs. Hypoxia (H)	
ko00232	caffeine metabolism	xanthine dehydrogenase/oxidase-like; xanthine dehydrogenase; uricase-like

## Data Availability

The data presented in this study are openly available in NCBI under BioProject number SUB12304423.

## Supplementary Materials

The following supporting information can be downloaded at https://www.mdpi.com/article/10.3390/antiox13091063/s1, Table S1: DEGs in the H vs. N comparison group; Table S2: DEGs in the R vs. N comparison group; Table S3: DEGs in the R vs. H comparison group.
